# LRRK2 G2385R and R1628P Mutations Are Associated with an Increased Risk of Parkinson's Disease in the Malaysian Population

**DOI:** 10.1155/2014/867321

**Published:** 2014-08-28

**Authors:** Aroma Agape Gopalai, Shen-Yang Lim, Jing Yi Chua, Shelisa Tey, Thien Thien Lim, Norlinah Mohamed Ibrahim, Ai Huey Tan, Gaik Bee Eow, Zariah Abdul Aziz, Santhi Datuk Puvanarajah, Shanthi Viswanathan, Irene Looi, Soo Kun Lim, Li Ping Tan, Yip Boon Chong, Chong Tin Tan, Yi Zhao, E. K. Tan, Azlina Ahmad-Annuar

**Affiliations:** ^1^Department of Biomedical Science, Faculty of Medicine, University of Malaya, 50603 Kuala Lumpur, Malaysia; ^2^Divisions of Neurology and Nephrology, Department of Medicine, Faculty of Medicine, University of Malaya, 50603 Kuala Lumpur, Malaysia; ^3^Department of Neurology, Hospital Pulau Pinang, 10990 Penang, Malaysia; ^4^Division of Neurology, Department of Medicine, Hospital Universiti Kebangsaan Malaysia, 56000 Kuala Lumpur, Malaysia; ^5^Division of Neurology, Department of Medicine, Hospital Sultanah Nur Zahirah, 20400 Kuala Terengganu, Malaysia; ^6^Department of Neurology, Hospital Kuala Lumpur, 50586 Kuala Lumpur, Malaysia; ^7^Department of Medicine, Hospital Seberang Jaya, 13700 Penang, Malaysia; ^8^Department of Clinical Research and Neurology, Singapore General Hospital, Singapore 169608

## Abstract

The *LRRK2* gene has been associated with both familial and sporadic forms of Parkinson's disease (PD). The G2019S variant is commonly found in North African Arab and Caucasian PD patients, but this locus is monomorphic in Asians. The G2385R and R1628P variants are associated with a higher risk of developing PD in certain Asian populations but have not been studied in the Malaysian population. Therefore, we screened the G2385R and R1628P variants in 1,202 Malaysian subjects consisting of 695 cases and 507 controls. The G2385R and R1628P variants were associated with a 2.2-fold (*P* = 0.019) and 1.2-fold (*P* = 0.054) increased risk of PD, respectively. Our data concur with other reported findings in Chinese, Taiwanese, Singaporean, and Korean studies.

## 1. Introduction

Parkinson's disease (PD) is an age-related illness, and, as populations age, the proportion of people with this neurodegenerative disease will continue to rise. It is projected that, by the year 2030, 9.3 million individuals above the age of 50 will suffer from PD and these cases will be concentrated outside the western world [1]. Studies have implicated exposure to environmental toxins and trauma as aetiological factors for PD [2]. Genetic variations also play a role, especially in cases where there is a family history of PD, which account for around 10–20% of all PD cases [3]. However, studies have shown that even late-onset sporadic PD may also have a genetic contribution [4].

One of the genes commonly implicated in both familial and sporadic PD is the leucine-rich repeat kinase 2 (*LRRK2*) gene. Several variants of* LRRK2* such as R1441C, G2019S, and I2020T have been well established as risk factors for PD [3]. Interestingly, there appear to be population-specific variants in LRRK2; for example, the G2019S variant is prevalent among the Ashkenazi Jews and North African Arabs (occurring in approximately 20% and 40% of PD patients in these groups, respectively [5]) but is absent in Asian populations (Chinese, Indian, Korean, and Japanese) [6, 7]. In Asian (Chinese, Taiwanese, Singaporean, and Japanese) populations, the G2385R variant is a more established risk variant but conversely is not found in Caucasian or Jewish patients with PD [8–12]. The R1628P is another common risk variant in Asian PD populations (Chinese, Taiwanese, and Singaporean) [13].

Given the lack of data regarding how these variants contribute to PD in Malaysian patients, we sought to investigate the prevalence of G2385R and R1628P in a Malaysian PD cohort. We found that G2385R was significantly associated with PD and R1628P showed a trend towards being a risk factor.

## 2. Methodology

A total of 1,202 subjects participated in this study. Six hundred and ninety-five PD patients were diagnosed by neurologists based on the United Kingdom PD Brain Bank Criteria and 507 controls who did not suffer from any neurological or movement disorders were recruited. Ethics approval and written consent from subjects were obtained. DNA was extracted from lymphocytes that were obtained from venous blood using the phenol-chloroform method. The G2385R (rs34778348) and R1628P (rs33949390) genotyping was done by Taqman allelic discrimination assay on a 7500 Fast Real-Time PCR machine. A subset of 20 individuals was sequenced to determine the error rate. The allele and genotype frequencies in PD cases and controls were compared with Fisher's exact test. Statistical analyses were performed using an open-source software (OpenEpi).

## 3. Results and Discussion

The mean age at PD diagnosis was 57.4 ± 11.8 years and the mean age of controls was 59.3 ± 9.4 years. Sixty percent of PD patients and 51% of controls were male. Results of the G2385R and R1628P genotyping are summarised in Table 1. The error rate of the assay was 0% in the subset of 20 individuals. Fifty-five patients (7.9%) had early-onset PD (onset < 40 years). Four patients were compound heterozygous for G2385R and R1628P; two of these patients had a family history of PD and developed PD before the age of 50, while the other two patients had no family history and had a later age of onset (>55).

The G2385R variant was associated with PD, with an odds ratio (OR) of 2.22 (*P* = 0.019), while the R1628P variant had an OR of 1.23 with a trend towards significance (*P* = 0.054). Interestingly, the G2385R mutation was present in control subjects as well (MAF = 0.001), although it was less frequently present than in the PD cohort (MAF = 0.026).

Our findings are in keeping with other published reports on G2385R, where this variant is associated with an increased risk of developing PD by approximately twofold (Chinese, Taiwanese, Singaporean, and Japanese populations) (Table 2). The G2385R variant is located within the WD40 domain of* LRRK2*, which is responsible for a variety of functions including signal transduction, pre-mRNA processing, and cytoskeleton assembly, and cells carrying the G2385R variant are more susceptible to oxidative stress and apoptosis [14].

The R1628P variant was first identified by Mata et al. [15]. Subsequently, Ross et al. reported this variant to be the second common genetic risk factor for PD in the ethnic Chinese (Taiwanese and Singaporean) population, with an OR of 1.84 (*P* = 0.006) [13]. Other independent studies carried out by Tan et al., Pulkes et al., and Lu et al. in Singapore, Thailand, and China showed a similar trend with OR values of 2.5, 3.3, and 2.1, respectively [16–18]. However, this was not observed in a Japanese cohort where the locus was found to be monomorphic [7]. This mutation alters a highly conserved amino residue within the “COR” domain of the LRRK2 protein [18]. The substitution of a highly basic polar arginine (R) with a neutral nonpolar proline (P) is likely to cause a conformational change in the protein secondary structure, thus altering the function of the protein. We note however that a recent multicentre study by Ross et al. involving 1386 Asian PD cases and 982 Asian controls did not find an association with R1628P (OR 0.62, 95% CI 0.36–1.07, *P* = 0.087) [19]. Whilst the findings in their Japanese and Korean subsets were consistent with previously published data, their Taiwanese cohort did not show a risk association, but rather a trend in the opposite direction (i.e., protective, with an OR of 0.56, 95% CI 0.32–1.01, *P* = 0.054).

In conclusion, our data concur with other reports in the Chinese, Taiwanese, Singaporean, and Korean populations. The G2385R variant is significantly associated with an increased risk of developing PD, while the R1628P variant is predicted to have a more modest effect. These data together with others can lead to a better understanding of the pathogenetic pathways leading to cell dysfunction and death in PD, with the ultimate hope that more specific drugs can be developed to treat this disabling disease.

## Figures and Tables

**Table 1:** Summary of the genotyping data.

SNP	PD (MAF)	Controls (MAF)	OR (95% confidence interval)
G2385R (c.7153G>A), rs34778348			
Wild type (G)	1354 (0.974)	1002 (0.999)	OR 2.22 (1.15–4.29)
Variant (A)	36 (0.026)	12 (0.001)	*P* = 0.019

R1628P (c.4883G>C), rs33949390			
Wild type (G)	1347 (0.969)	996 (0.982)	OR 1.23 (1.039–1.448)
Variant (C)	43 (0.031)	18 (0.018)	*P* = 0.054

**Table 2:** Summary of published Asian data on G2385R and R1628P.

Study	Asian country	Sample size	Results
G2385R (c.7153G>A), rs34778348			
Di Fonzo et al., 2006 [9]	Taiwan	608 PD, 373 controls	OR 2.24 (*P* = 0.004)

Fung etal., 2006 [20]	Taiwan	305 PD, 176 controls	OR 17.00 (*P* = 0.0002)

Farrer et al., 2007 [21]	Taiwan	410 PD, 335 controls	OR 2.24 (*P* = 0.014)
Tan et al., 2007 [14]	Singapore	495 PD, 494 controls	OR 2.14 (*P* = 0.014)

Tan et al., 2007 [16]	Non-Chinese Asian (Malays and Indians)	98 PD, 173 controls 66 PD, 133 controls	Malays OR 1.78 (*P* = 0.3) Indians-monomorphic

An et al., 2008 [11]	Mainland China	600 PD, 334 controls	OR 3.94 (*P* < 0.01)

Funayama et al., 2007 [10]	Japan	448 PD, 457 controls	OR 2.60 (*P* = 1.24 × 10^−4^)

Zabetian et al., 2009 [7]	Japan	601 PD, 1,628 controls	OR 1.96 (*P* < 0.001)

Miyake et al., 2010 [22]	Japan	229 PD, 358 controls	OR 2.06

Kim et al., 2010 [12]	Korea	923 PD, 422 controls 119 YOPD 814 LOPD	Combined OR 1.83 (*P* = 0.017) YOPD OR 2.28 (*P* = 0.098) LOPD OR 1.81 (*P* = 0.022)

Ross et al., 2011 [19]	Asian	Taiwanese 369 PD, 300 controls Korean 844 PD, 587 controls Japanese 173 PD, 95 controls Combined 1,386 PD, 982 controls	OR 1.62 ∗*P* value not stated OR 1.87 ∗*P* value not stated OR 1.44 ∗*P* value not stated OR 1.73 (*P* = 0.0026)

*Current study *	Malaysia	695 PD, 507 controls	OR 2.22 (*P* = 0.019)

R1628P(c.4883G>C), rs33949390			
Mata et al., 2005 [15]	Europe, Asia, and North America	100 PD probands with family history of parkinsonism, 300 controls	MAF 0.01

Lu et al., 2008 [18]	Taiwan	834 PD, 543 controls	OR 2.13 (*P* = 0.004)

Tan et al., 2008 [16]	Singapore	246 PD, 243 controls	OR 2.5 (*P* = 0.046)

Tan et al., 2008 [23]	Non-Chinese Asian (Malays and Indians)	132 PD, 160 controls 60 PD, 105 controls	OR 0.61 (*P* = 0.600) Indians-monomorphic

Ross et al., 2008 [13]	Taiwan, Singapore	Wu RM 484 PD, 341 controls Wu YR 345 PD, 316 controls EK Tan 250 PD, 250 controls Combined 1079 PD, 907 controls	OR 2.15 (*P* = 0.025) OR 1.39 (*P* = 0.179) OR 2.20 (*P* = 0.163) OR 1.84 (*P* = 0.006)

Zabetian et al., 2009 [7]	Japanese	631 PD, 320 controls	Monomorphic

Yu et al., 2009 [24]	Mainland China	328 PD, 300 controls	OR 2.68 (*P* < 0.05)

Zhang et al., 2009 [25]	Mainland China	600 PD, 459 controls	OR 3.14 (*P* < 0.01)

Kim et al., 2010 [12]	Korea	384 PD, 384 controls	OR 2.98 (*P* = 0.32)

Pulkes et al., 2011 [17]	Thai	154 PD, 156 controls	OR 3.25 (*P* = 0.021)

Ross et al., 2011 [19]	Asian	Taiwanese (369 PD, 300 controls)	OR 0.56 (*P* = 0.054)
		Korean (844 PD, 587 controls)	OR 2.47 (*P* = 0.42)
		Japanese (173 PD, 95 controls)	Monomorphic
		Combined (1,386 PD, 982 controls)	OR 0.62 (*P* = 0.087)

*Current study *	Malaysian	695 PD, 507 controls	OR 1.23 (*P* = 0.054)
